# Efficacy of an Essential Oil-Based Pesticide for Controlling Bed Bug (*Cimex lectularius*) Infestations in Apartment Buildings

**DOI:** 10.3390/insects5040849

**Published:** 2014-11-05

**Authors:** Changlu Wang, Narinderpal Singh, Richard Cooper

**Affiliations:** Department of Entomology, Rutgers University, New Brunswick, NJ 08901, USA; E-Mails: nsingh@aesop.rutgers.edu (N.S.); rcooper@aesop.rutgers.edu (R.C.)

**Keywords:** *Cimex lectularius*, integrated pest management, essential oils, efficacy

## Abstract

Bed bugs (*Cimex lectularius* L. and *Cimex hemipterus* F.) are among the most difficult urban pests to manage. Many essential oil-based bed bug control products that are considered reduced risk to mammals compared to synthetic insect neurotoxins have become commercially available, but their effectiveness as a stand-alone control method is unknown. This study assessed the field efficacy of an essential oil-based bed bug control product (EcoRaider; a.i. 1% geraniol + 1% cedar oil + 2% sodium lauryl sulfate) compared to a pyrethroid and neonicotinoid mixture spray (0.075% Temprid SC; a.i. beta-cyfluthrin + imidacloprid). After 12 weeks, the three treatments—EcoRaider, Temprid SC, and EcoRaider + Temprid SC caused 92.5 ± 2.7, 92.9 ± 3.0, and 91.7% ± 2.7% bed bug count reduction, respectively. No significant differences existed in the bed bug reduction among the treatments. Bed bugs were eliminated from only 22% of the treated apartments. Among those still with bed bugs, 76% of the residents did not know bed bugs were present. We documented the residents’ self-control practices and discussed the potential of using essential oil-based insecticides in bed bug management programs to minimize the health risks to building occupants and pets and to slow down the development of insecticide resistance.

## 1. Introduction

The resurgence of bed bugs, *Cimex lectularius* L. and *Cimex hemipterus* F., in recent years triggered the development of many insecticide products for bed bug control in the USA. According to United States Environmental Protection Agency (EPA) [1], the registered active ingredients for bed bug control include 16 pyrethrins and pyrethroids, 4 neonicotinoids, 3 inorganic compounds, chlorfenapyr, DDVP (dichlorvos), propoxur, S-hydroprene, alcohol, and neem oil. The majority are pyrethroids, which have limited field efficacy due to wide spread resistance in field bed bug populations [2,3]. Pest management firms primarily use pyrethroids, chlorfenapyr, mixtures of a pyrethroid and a neonicotinoid insecticide, and various silicate-based insecticides such as diatomaceous earth dust to control bed bugs [4]. Besides EPA registered products, dozens of minimal risk pesticide products that fall under the Federal Insecticide, Fungicide, and Rodenticide Act’s Section 25(b) have become commercially available [5]. These products (hereafter referred to as “25(b)”) contain one or several of the following 16 active ingredients: cedar oil, cinnamon oil, citric acid, citronella oil, clove oil, eugenol, geraniol, geranium oil, lauryl sulfate, lemongrass oil, peppermint oil, rosemary oil, 2-Phenethyl propionate, potassium sorbate, sodium chloride, and sodium lauryl sulfate. These products are not subject to efficacy data requirements by EPA as are the rest of the pesticides.

Hiring professionals to control a bed bug infestation is expensive [4]. Thus, homeowners commonly use pesticides themselves to avoid paying the cost of professional pest control service. Unfortunately, the numbers of registered bed bug control products available to the public are more limited than those for professionals with a pesticide application license. All of the synthetic consumer products sold in the USA belong to pyrethrins or pyrethroids, plus the silicate insecticides are also widely available. Furthermore, the efficacy of the consumer products labeled for bed bugs is questionable given the fact that most field bed bug populations examined currently exhibit moderate to high levels of resistance to pyrethroids [3]. For instance, insect foggers commonly used by consumers have little effect on field collected bed bugs [6]. In our laboratory studies, two commonly used bed bug sprays, 0.03% lambda-cyhalothrin (Hot Shot Bed Bug and Flea Killer; Spectrum Group, St. Louis, MO, USA) and 0.4% phenothrin + 1.6% MGK-264 (Pronto Plus Kill Bedbugs and Dust Mites; Insight Pharmaceuticals Corp., Langhorne, PA, USA) caused 0% mortality when bed bugs from a field strain were sprayed directly (Appendix 1). Although the silicate based products have demonstrated excellent efficacy in most published laboratory trials, their field efficacy is unknown. Lack of efficacy of the available bed bug control products leads to chronic infestations, frequent treatments, use of off-label products by consumers or even spread of the infestation into adjoining premises in multiple occupancy dwellings [7,8].

Bed bugs commonly hide on beds and upholstered furniture for the convenience of easy access to the host [9,10,11]. Ninety-four percent of pest management professionals surveyed reported applying pesticides to beds for the control of bed bugs [4]. Applying insecticides to these areas creates opportunities for human insecticide exposure. Therefore, consumers as well as professionals have strong interests in insecticides that have minimal health risks to humans and pets. Dozens of 25(b) products have become available in response to the need for safe treatment of bed bug infested furniture. They are commonly used by consumers who are suffering bed bug infestations. Despite their popularity, there is only one scientific report documenting the efficacy of 25(b) bed bug sprays [12]. Among the 11 25(b) products tested, only EcoRaider (1% geraniol + 1% cedar oil + 2% sodium lauryl sulfate; Reneotech, North Bergen, NJ, USA) and Bed Bug Patrol (0.03% clove oil + 1% peppermint oil + 1.3% sodium lauryl sulfate; Nature’s Innovation, Buford, GA, USA) caused >90% mortality. Nine of the 11 tested products caused 0%–61% mortality in direct spray laboratory assays. There are no field efficacy data on 25(b) products. It is apparent that there is a knowledge gap regarding the role of essential oil-based products in bed bug management.

In this study, our objectives are: (1) to determine the effectiveness of EcoRaider spray in naturally infested apartments; and (2) to determine whether EcoRaider alone or in combination with a synthetic insecticide will provide a similar level of control compared with synthetic insecticide alone. Temprid SC (21% imidacloprid + 10.5% beta-cyfluthrin; Bayer Environmental Science, Research Triangle Park, NC, USA) was selected for comparison in this study. It is a commonly used synthetic insecticide for bed bugs by pest management professionals. Previous evaluations show this is a highly effective synthetic insecticide for bed bugs [8,12].

## 2. Experimental Section

### 2.1. Study Site and Selection of Test Apartments

The study was conducted in two high-rise apartment buildings consisting of a total of 409 occupied apartments located at Irvington, NJ, USA. Among them, 37% were one bed room apartments and the rest were studio apartments. Most of the apartments were occupied by senior citizens and did not have air conditioners in use during the study period. The bed bug infestations were controlled by residents themselves or a housing staff using insecticide sprays and diatomaceous earth dust. Bed bugs were collected from 5 apartments about 8 months prior to the study. Direct spray with the label rate of Suspend SC (0.06% deltamethrin; Bayer Crop Science, Research Triangle Park, NC, USA) at 4.07 mg/cm^2^ yielded 60.0% ± 7.7% mortality after 7 days (based on three replicates, 15 bed bug nymphs per replicate). Therefore, we considered the bed bugs in the two buildings to have low to medium level resistance to pyrethroids.

We first identified bed bug infested apartments based on infestation records from the housing staff. Climbup insect interceptors (Susan McKnight Inc., Memphis, TN, USA) were placed under the legs of beds and upholstered furniture (or beside the furniture legs if the legs were too large) in these apartments and examined after two weeks to obtain pre-treatment bed bug counts [13]. If the furniture did not have legs, the interceptors were placed beside the corners of the furniture. Twenty four apartments with 9–318 bed bugs based on interceptor counts (23 apartments) or a combination of interceptor counts and a thorough visual inspection (one apartment) were selected. We used total count from interceptors and a visual inspection from one apartment because the interceptors only caught three bed bugs and a visual inspection found 23 bed bugs before treatment. The interceptors were placed beside the infested sofa and couch due to lack of furniture legs in this apartment. Among the 24 selected units, 14 residents used insecticide sprays (13 used pyrethrins/pyrethroids and one used essential oils), seven residents installed electronic pest repellers to reduce insects and rodents prior to our study, seven residents did not use any pest control products, 21 of the selected units had beds, of which 17 encased their mattresses and box springs in plastic (15 apartments) or fabric (two apartments). We asked the residents to stop using insecticides. The 24 apartments were divided into three similar groups (eight apartments each) based on bed bug counts.

### 2.2. Treatments

Apartments in each group were randomly assigned to one of three treatments: I—EcoRaider, II—Temprid SC, or III—combination of EcoRaider and Temprid SC which were applied using separate spray tanks to different locations within apartments. Both EcoRaider and Temprid SC were provided by the manufacturers. Each group received only one of the three treatments. The sprays were applied using B and G compressed air sprayers (Univar Inc., Edison, NJ, USA) following the product label instructions. Temprid SC was diluted to 0.075% (imidacloprid + beta-cyfluthrin) with water following the label directions. A comprehensive spray treatment was performed in each of the test apartments at 8–10 days after the pre-count was obtained. The initial treatments were conducted between 17 September 2013 and 19 September 2013. In treatments I and II, the product was applied to bed bug harborages on furniture (mattress and box spring, sofa, chair, and luggage), perimeters of the floors, cracks on walls near beds and furniture, as well as along curtain folds and wall decorations if bed bug activity was observed. In treatment III, Temprid SC was applied to cracks and crevices of room perimeter, and bottom of sofas. EcoRaider was applied on furniture, curtains and wall decorations where bed bug activity was noticed. We used EcoRaider in these areas because Ecoraider had more liberal label directions for application to beds and upholstered furniture. Interceptors were installed under bed and sofa legs or beside the furniture legs immediately after the treatment to monitor the effectiveness of the treatments. The initial treatment time and material use in each apartment were recorded.

Follow-up visits were conducted biweekly during which a brief visual inspection of beds and upholstered furniture was conducted to guide additional treatments. The sofas were turned over to examine for bed bugs. The mattress and box springs were turned over only if they were not encased or any bed bugs were found in interceptors. Additional sprays were applied only to harborages where live bed bugs were found for all three treatment groups.

In all treatment groups, residents were provided with a brochure about how to recognize bed bugs, how to prevent getting bed bugs, and both non-chemical and chemical control methods. Residents were encouraged to launder their bed sheets and clothing frequently. Five residents discarded furniture or medical equipment, two residents installed mattress encasements, and one resident purchased a metal bed frame to replace a broken bed frame. These procedures assisted in reducing bed bug numbers. Two thirds of the apartments remained cluttered and four residents rarely washed their bed sheets during the study. We did not include an un-treated control because residents would be reluctant to participate in the study if their apartments were left un-treated for an extended period of time while being visited periodically by our research team.

### 2.3. Post-Treatment Monitoring

The interceptors installed under the bed and sofa legs were inspected at 2, 4, 6, 9, and 12 weeks post-initial treatment. If bed bugs were not found in interceptors, a thorough visual inspection of the beds, upholstered furniture, and the surrounding areas was conducted to confirm bed bug elimination. During each visit, we interviewed residents whenever possible to record whether they noticed bed bug activity. All apartments (except one due to lack of access) were thoroughly inspected at 12 weeks.

### 2.4. Statistical Analysis

The bed bug counts from interceptor catches and/or visual inspections were logarithmic transformed prior to analysis. The 0 week transformed count data were subject to Analysis of Variance to determine if there are significant differences in initial bed bug counts among treatments and among apartments with or without pest repellers. The 0 to 12 week transformed count data were analyzed using the PROC MIXED procedure in SAS software to determine whether there were significant differences in the speed and level of bed bug reduction among the three treatments [14]. Paired *t*-test was used to analyze the differences in the amount of insecticides used by residents before the study and amount of insecticides used by researchers in this study. The mean service time in each apartment at each monitoring period and cumulative pesticide use per apartment was compared among the three treatments using PROC GLM. Means were separated by Fisher’s LSD test.

## 3. Results

The initial mean (±SEM) bed bug counts in I, II, and III treatment groups were 72 ± 36, 63 ± 29, and 58 ± 18, respectively. The median (min, max) bed bug counts in the three treatment groups were: 38 (9, 318), 31.5 (10, 253), and 38.5 (11, 170), respectively. There were no significant differences in the initial bed bug counts among treatment groups (F = 0.03; df = 2, 21; *p* = 0.97).

**Figure 1:**
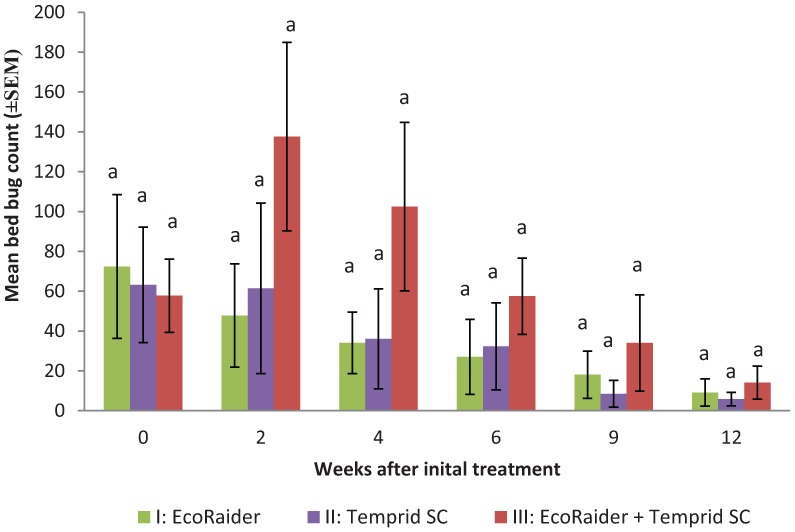
Bed bug counts in apartments before and after three different treatments. Means within the same observation period with same letters are statistically not different (ANOVA, *p* > 0.05).

**Figure 2:**
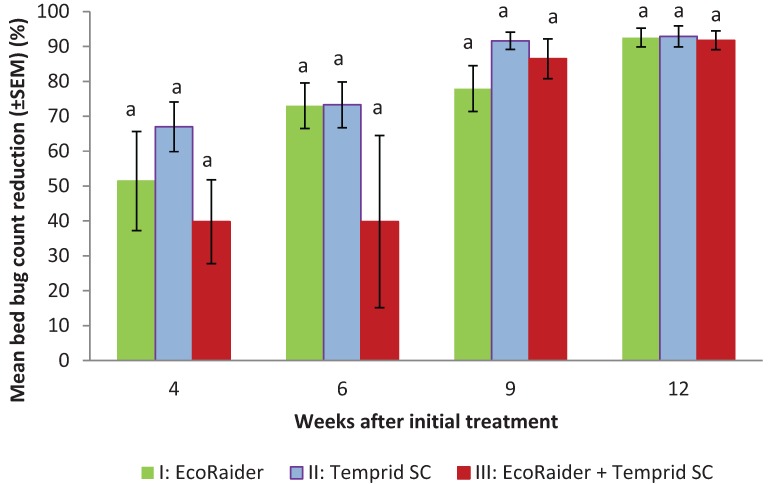
Effect of three treatments on bed bug count reduction. Means within the same observation period with same letters are statistically not different (ANOVA, *p* > 0.05).

At 2 weeks, the bed bug counts from the three treatments did not decline as expected, especially in treatment III with the average count increased from 58 to 138 (Figure 1). This was most likely due to the inherent limitation of using interceptors alone for estimating bed bug populations. We therefore used either the 0 or 2 week count whichever was greater as the base count for calculating bed bug count reduction at 4, 6, 9, and 12 weeks. The bed bug counts declined in all groups significantly during 4 to 12 weeks (F = 4.2; df = 11, 59; *p* = 0.0001) (Figure 2). There were no significant differences in bed bug count reduction among the three treatments at each observation period (*p* > 0.05). At 12 weeks, the mean bed bug counts in treatments I, II, and III were: 9.1 ± 6.8, 5.9 ± 3.4, and 14.1 ± 8.3, respectively (Figure 1). Mean bed bug count reduction in the three treatments was 92.5 ± 2.7, 92.9 ± 3.0, and 91.7% ± 2.7%, respectively (Figure 2).

**Figure 3:**
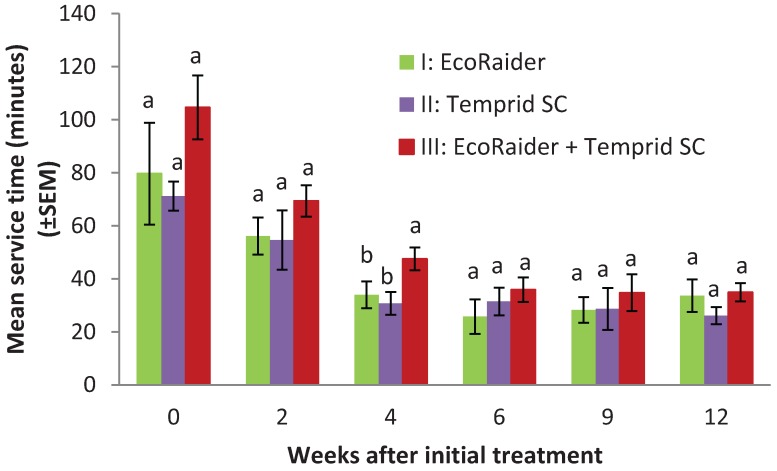
Mean service time per apartment for three different treatments. Means within the same observation period with different letters are statistically different (Fisher’s LSD test, *p* < 0.05).

At 12 weeks, bed bugs could no longer be found in two apartments from treatment I, two apartments from treatment II, and one apartment from treatment III, determined by interceptors, visual inspections, and interviews with residents. These five apartments also had the lowest count (range 9–14) prior to the treatment. A total of 18 (78%) apartments (one apartment in treatment II was not accessed) still had bed bugs based on interceptor counts (mean: 11; range: 1–70). Visual inspections only found bed bugs in 50% of the infested apartments. Interviews with 17 residents whose apartments still had bed bugs show that 76% (13 residents) of them did not feel bites or see bed bugs. Mean bed bug count in these 13 apartments based on 2 week interceptor placement was 11. Two to three researchers serviced each apartment during each visit. The technician time (mean ± SEM) spent per apartment during the initial treatment for treatment I, II, and III were 80 ± 19, 71 ± 5, and 105 ± 12 minutes, respectively (Figure 3). The treatment time were not significantly different (F = 1.4; df = 2, 19; *p* = 0.27). Similarly, there were no significant differences in the service time during the follow-up visits at 2 weeks (F = 0.94; df = 2, 21; *p* = 0.41), 6 weeks (F = 0.87; df = 2, 21; *p* = 0.44), 9 weeks (F = 3.3; df = 2, 19; *p* = 0.72), and 12 weeks (F = 1.0; df = 2, 20; *p* = 0.38). However, the treatment III required significantly longer time than the other two groups at 4 weeks due to higher bed bug counts than the other two groups (F = 3.8; df = 2, 21; *p* = 0.04).

The amount of pesticide used per apartment in I, II, and III treatments during week 0–9 were 0.97 ± 0.37, 0.89 ± 0.22, and 1.59 ± 0.31 L, respectively. They were not significantly different (F = 1.59; df = 2, 21; *p* = 0.23). In treatment III, an average of 1.07 ± 0.24 and 0.52 ± 0.10 L of EcoRaider and Temprid SC were used, respectively. Significantly more EcoRaider was used than Temprid SC in this treatment because the protocol specifies that only EcoRaider would be applied to live bed bugs found on beds and upholstered furniture (*t* = 2.78; df = 7; *p* = 0.03) where visible bed bugs were often present.

We interviewed 18 residents regarding their pesticide use prior to our treatments. A total of 16 (89%) residents used at least one type of consumer products for bed bug control. The leading (13 residents) type of self-control products was pyrethroid sprays (liquid sprays or aerosol sprays). Ten out of the 18 residents provided information on the amount of insecticide sprays used to control bed bugs. Among them, one resident applied approximately 38 L (10 gal) of spray over 12 month period. The other nine residents applied an average of 3.56 ± 0.48 L of spray before our treatment. In these nine units, we used an average of 1.68 ± 0.30 L insecticide spray, which was significantly less than that used by residents (*t* = −2.89; df = 8; *p* = 0.02). The following self-control products were also used by residents: silicon dioxide dust, essential oils, rubbing alcohol, and household cleaning sprays. Two insecticide sprays not registered for bed bug control were used by residents: 0.05% bifenthrin + 0.015% zeta-cypermethrin (Ortho Home Defense Max Insect Killer; The Ortho Group, Marysville, OH, USA) and 0.1% imiprothrin + 0.1% cypermethrin (Raid Ant and Roach aerosol; S.C. Johnson and Son Inc., Racine, WI, USA).

Among the 24 infested apartments, six had electronic pest repellers in use for repelling pests. The types of repellers included Bell and Howell Electromagnetic/Ultrasonic Pest Repeller (model SB-104) [15], Riddex Sonic Plus (Global TV Concepts, Van Nuys, CA, USA), and Poison-free pest chaser (model M690/M692) (Woodstream Littitz, PA, USA). Five of the residents who installed pest repellers also used pyrethroid sprays. The mean bed bug count in apartments with pest repellers (105.8 ± 48.6) was not significantly different from those without repellers (49.3 ± 13.6) (F = 0.7; df = 1, 22; *p* = 0.41) prior to the treatments.

## 4. Discussion

Homeowners commonly use insecticides themselves when they find pests at home [16]. With the resurgence of bed bugs, many sprays, aerosols, and foggers labeled for bed bugs have become widely available. Among these products, pyrethroids are the dominant insecticide class of consumer insecticide products found in stores and in homes. This study revealed that high percentage (89%) of interviewed residents who suffered bed bug infestations used insecticides to control bed bugs themselves. In addition to the prevalence of self-application of insecticides in homes, consumers tend to over-apply insecticides [17]. For instance, one resident used 38 L of 0.03% lambda-cyhalothrin (Hot Shot Bed Bug and Flea Home Insect Killer) over 12 months. Another resident used 50 cans (496 g per can) of 0.4% phenothrin + 1.53% MGK 264 (Raid Max Bed Bug and Flea Killer aerosol; S.C. Johnson and Son Inc., Racine, WI, USA) over three years. The excessive and frequent introduction of insecticides into the apartments would likely leave high levels of residue in the environment. Furthermore, the application is usually targeted to beds, sofas, or other furniture where residents sleep or rest and bed bugs hide, creating opportunities of insecticide exposure in these areas. A recent study shows the concentration of pyrethroids and pyrethroid metabolites in floor wipe samples is correlated with urinary concentrations of pyrethroid metabolites [18]. Additional studies on the relationships among the frequency of insecticide use on furniture, the insecticide residue levels on furniture surfaces, and concentrations in human body (such as urine or blood) may be helpful in estimating exposure and potential health impacts from self-control efforts by residents.

Because of the prevalence of consumer misuse of insecticides and associated health concerns, identifying low-risk and effective alternative insecticides will have immediate benefit to consumers who wish to control bed bugs themselves. The comparable performance of the essential oil-based spray with the conventional insecticide spray demonstrated that non-synthetic materials may be used to reduce pyrethroid residues in homes. The ingredients in EcoRaider are relatively safer to humans and pets compared to insect neurotoxins based on U.S. EPA’s classification of pesticides. Using an effective essential oil-based product is most advantageous in sensitive environments such as classrooms, day care centers, prisons, and hospitals. In addition, essential oil-based products could provide an effective alternative for managing insecticide resistant populations, which are widespread in the U.S. Currently, the available chemical sprays in the U.S. for managing bed bugs are limited to pyrethrins, pyrethroids, chlorfenapyr, pyrethroid + neonicotinoid mixtures.

Ecoraider has a noticeable smell that can last for more than two weeks after application. Although none of the residents that received EcoRaider treatment complained about the smell, it is advisable that users should be aware of this when considering the use of this product. Like many other essential oil-based insecticide products, EcoRaider is much more expensive than the commonly used synthetic insecticide sprays. A gallon (3.785 L) of EcoRaider costs $89.95 and a 16 oz (473.2 mL) bottle costs $19.95 [19]. In comparison, the popular consumer bed bug control spray, Hot Shot Bed Bug and Flea Killer, costs $9.97 per gallon [20]. A gallon of diluted pyrethroid spray or mixture of pyrethroid and neonicotinoid used by professionals costs only a few dollars. Despite the higher cost of EcoRaider compared to pyrethroid sprays, for those who cannot afford the cost of professional pest management services, it provides an affordable alternative for consumers. However, users should educate themselves and learn where bed bugs hide and target the treatment to bed bug harborages to maximize the effectiveness of the treatment and minimize misuse.

An interesting note is that the active ingredients in EcoRaider are not unique. For example, geraniol and cedar oil are present in Bed Bug Fix (NuSafe Floor Solutions, Walton, KY, USA). Geraniol and sodium lauryl sulfate are present in Rest Assured (ES and P Global, Miami, FL, USA). However, the efficacy of these two products against bed bugs is substantially lower than EcoRaider. Both Bed Bug Fix and Rest Assured caused <50% mortality against bed bugs, whereas, EcoRaider caused 100% mortality to bed bugs under laboratory conditions [12]. According to the EcoRaider manufacturer, their product is micro-emulsified and their cedar oil (trade secret) may be more effective than the cedar oil used in other essential oil-based products. Another possible factor is the interactions among the active ingredients which enable the active ingredients to more effectively penetrate the insect cuticles. Further research on the relative toxicity of essential oils and their interactions will be instrumental in developing more effective products.

In spite that relatively more effective products were tested in this study, the sprays alone did not eliminate most of the bed bug infestations after four follow-up inspections and re-treatments (at 2, 4, 6, and 9 weeks), further confirming the limitations of insecticide treatments in eliminating bed bug infestations [21]. The fact that all of the five apartments with successful bed bug elimination also had the lowest bed bug count at the beginning (range 9–14) suggests that the higher the infestation level, the longer time is needed for elimination. Lack of resident cooperation is one contributing factor. Most of the residents were not bothered by the presence of bed bugs even when bed bugs were still present at 12 weeks. Presence of clutter and infrequent laundering of the bed sheets allow some bed bugs to be left unexposed to pesticides during treatment because sprays cannot be applied on these items. Therefore, part of a successful bed bug elimination campaign should include a plan to remove these obstacles. There are many non-chemical tools and methods available that are both safe and effective [10,22,23,24,25]. These include reduction of harborages through de-cluttering and sealing harborages, frequent laundering and drying, vacuuming, heat, and cold. Selectively incorporating these methods based on the characteristics of the infestation, environmental conditions, and residents’ behavior will no doubt provide faster elimination of bed bugs and further reduce the need for insecticide applications.

## 5. Conclusions

Although many of the non-synthetic insecticides failed to provide good control in laboratory studies, the current study showed one product resulted in bed bug reduction that is comparable to that by a synthetic insecticide spray. The low-risk non-synthetic spray may be especially suitable for use by untrained consumers who tend to over apply insecticides and apply insecticides to exposed surfaces that are likely to cause human exposure. Expedient elimination requires incorporation of multiple control measures and resident cooperation.

## Appendix 1. Efficacy of Two Pyrethroid Insecticides against Bed Bugs.

Two consumer products, Hot Shot Bed Bug and Flea Killer and Pronto Plus Kill Bedbugs and Dust Mites (Insight Pharmaceuticals Corp., Langhorne, PA, USA) were evaluated for their efficacy against a field strain bed bugs in a direct spray bioassay. Twenty 4–5th instar bed bug nymphs were used in each replication. Bugs were starved for 7 days before treatments. Bed bugs in the control group were sprayed with water. Each treatment was replicated three times. Bed bugs were transferred to clean petri dishes immediately after spray treatment and were kept at 25 °C in the laboratory. Both pesticides caused 0% mortality in bed bug nymphs at 3 days after treatment. No control mortality was observed.
